# Adoption of Wearable Insulin Biosensors for Diabetes Management: A Cross-Sectional Study

**DOI:** 10.7759/cureus.50782

**Published:** 2023-12-19

**Authors:** Turki M Alanzi, Wala Alzahrani, ‏Mohammed Almoraikhi, ‏Asayil Algannas, Mohammed Alghamdi, ‏Lujain Alzahrani, Ruba Abutaleb, ‏Renad Ba Dughaish, Nada Alotibi, Shayma Alkhalifah, ‏Mona Alshehri, Hayat Alzahrani, ‏Reham Almahdi, Nouf Alanzi, ‏Nesren Farhah

**Affiliations:** 1 Department of Health Information Management and Technology, College of Public Health, Imam Abdulrahman Bin Faisal University, Dammam, SAU; 2 Department of Clinical Nutrition, College of Applied Medical Sciences, King Abdulaziz University, Jeddah, SAU; 3 ‏College of Medicine, Imam Abdulrahman Bin Faisal University, Dammam, SAU; 4 ‏‏College of Medicine, Al Baha University, Al Baha, SAU; 5 Department of Pharmaceutical Services, Dhahran Long Term Care Hospital, Dhahran, SAU; 6 College of Medicine, ‏King Abdulaziz University, Jeddah, SAU; 7 College of Pharmacy, Jazan University, Jazan, SAU; 8 College of Medicine, Alfaisal University, Riyadh, SAU; 9 College of Pharmacy, Shaqra University, Shaqra, SAU; 10 College of Medicine, Imam Abdulrahman Bin Faisal University, Dammam, SAU; 11 ‏College of Medicine, Princess Nourah Bint Abdul Rahman University, Riyadh, SAU; 12 College of Pharmacy, Taif University, Taif, SAU; 13 ‏College of Medicine, Al Baha University, Al Baha, SAU; 14 Department of Clinical Laboratories Sciences, College of Applied Medical Sciences, Jouf University, Jouf, SAU; 15 Department of Health Informatics, College of Health Sciences, Saudi Electronic University, Riyadh, SAU

**Keywords:** trust, monitoring, biosensors, adoption, diabetes, insulin, artificial intelligence

## Abstract

Background: Wearable insulin biosensors represent a novel approach that combines the benefits of real-time glucose monitoring and automated insulin delivery, potentially revolutionizing how individuals with diabetes manage their condition.

Study purpose: To analyze the behavioral intentions of wearable insulin biosensors among diabetes patients, the factors that drive or hinder their usage, and the implications for diabetes management and healthcare outcomes.

Methods: A cross-sectional survey design was adopted in this study. The validated questionnaire included 10 factors (Performance expectancy, effort expectancy, social influence, facilitating conditions, behavioral intention, trust, perceived privacy risk, and personal innovativeness) affecting the acceptance of wearable insulin sensors. A total of 248 diabetic patients who had used wearable sensors participated in the study.

Results: Performance expectancy was rated the highest (Mean = 3.84 out of 5), followed by effort expectancy (Mean = 3.78 out of 5), and trust (Mean = 3.53 out of 5). Statistically significant differences (p < 0.05) were observed with respect to socio-demographic variables including age and gender on various influencing factors and adoption intentions. PE, EE, and trust were positively associated with adoption intentions.

Conclusion: While wearable insulin sensors are positively perceived with respect to diabetes management, issues like privacy and security may affect their adoption.

## Introduction

Diabetes is a multifaceted chronic condition, characterized by insufficient insulin production or impaired insulin utilization, resulting in elevated blood glucose levels [1]. Effective diabetes management involves maintaining blood glucose levels within a healthy range to prevent complications, which can include cardiovascular disease, kidney failure, neuropathy, and blindness [2-4]. Achieving glycemic control requires regular monitoring of blood glucose levels, careful dietary management, physical activity, and often the use of insulin or other antidiabetic medications [5,6]. According to the International Diabetes Federation (IDF), in 2021, globally, an estimated 537 million adults were living with diabetes, with the number projected to rise to 783 million by 2045 [7]. The management of diabetes is a complex and lifelong challenge, necessitating regular monitoring of blood glucose levels and precise insulin administration. While there have been substantial advancements in diabetes management over the years, there remains an ongoing need for innovative solutions to improve the lives of those living with diabetes and enhance their self-care capabilities.

Historically, self-monitoring of blood glucose (SMBG) has been the gold standard for managing diabetes. Individuals with diabetes have relied on glucometers to measure their blood glucose levels through periodic fingerstick tests. The data from these tests have informed their insulin dosages and helped them make necessary lifestyle adjustments. However, SMBG has several limitations [8,9]. It is an episodic and retrospective process, providing only intermittent insights into glucose levels. As a result, it may not capture fluctuations or sudden changes in blood glucose, making it challenging to maintain optimal glycemic control. Additionally, the need for frequent fingerstick tests can be painful and burdensome, potentially leading to non-compliance and suboptimal self-management [10].

Continuous glucose monitoring (CGM) systems, for example, use tiny sensors inserted beneath the skin to continuously measure interstitial glucose levels, providing a more comprehensive and real-time view of blood glucose fluctuations [11,12]. These systems can send data to a receiver or smartphone, alerting users to high or low glucose levels. They offer a more comprehensive understanding of glucose patterns, help individuals make informed decisions about insulin dosing and carbohydrate intake, and reduce the risk of hypoglycemic and hyperglycemic episodes. Insulin pumps, on the other hand, automate insulin delivery by continuously infusing insulin into the body, and they can be programmed to adjust insulin delivery based on real-time CGM data [13,14]. This integration of CGM and insulin delivery into a single device can help maintain more stable blood glucose levels, potentially reducing the burden of diabetes management.

Wearable insulin biosensors: a paradigm shift

While CGMs and insulin pumps have been significant innovations in diabetes care, the latest frontier is the development of wearable insulin biosensors that combine the benefits of both technologies. These biosensors have the potential to provide a seamless and automated solution for individuals with diabetes, allowing for continuous monitoring of glucose levels and the automatic administration of insulin when needed [13]. This paradigm shift from episodic self-monitoring and manual insulin dosing to continuous, real-time monitoring and automated insulin delivery has the potential to revolutionize diabetes management. Wearable insulin biosensors typically consist of a CGM component for CGM and an insulin delivery component, which can include an insulin pump or another mechanism for insulin administration. These devices can communicate wirelessly with a user's smartphone, providing real-time data and alerts, and allowing for remote monitoring and adjustments by healthcare providers and caregivers [14,15]. The potential advantages of wearable insulin biosensors are numerous [16-20]. 

Improved Glycemic Control

By providing continuous real-time glucose data and automated insulin delivery, wearable insulin biosensors have the potential to improve glycemic control and reduce the risk of hypoglycemia and hyperglycemia. This can result in better overall health outcomes and a reduced risk of diabetes-related complications.

Enhanced Quality of Life

The convenience and reduced burden of diabetes management through wearable insulin biosensors can significantly enhance the quality of life for individuals with diabetes. It reduces the need for frequent fingerstick tests and manual insulin dosing, allowing for more freedom and flexibility in daily life.

Increased Adherence

The automated nature of these devices can improve adherence to treatment plans, as they remove some of the cognitive and logistical challenges associated with diabetes self-care. This can lead to more consistent adherence to insulin regimens and lifestyle recommendations.

Remote Monitoring and Support

Wearable insulin biosensors can be integrated with telemedicine and remote monitoring systems, enabling healthcare providers and caregivers to support individuals with diabetes from a distance. This can be especially valuable in cases where individuals may require additional assistance or have complex diabetes management needs.

Personalized Care

These devices can collect extensive data on an individual's glucose patterns and insulin requirements, allowing for more personalized and precise diabetes management. By leveraging artificial intelligence (AI) and data analytics, wearable insulin biosensors can adapt and optimize insulin delivery based on an individual's unique needs.

Wearable insulin biosensors offer promising avenues for diabetes management but come with certain disadvantages and risks. These include potential technical issues such as inaccurate readings, calibration challenges, or device malfunctions, leading to incorrect insulin dosing. Skin irritation or allergic reactions at the sensor site pose concerns for some users [21]. Additionally, the reliance on a technological device introduces the risk of dependency, where users may overlook traditional monitoring methods or self-awareness of their condition. Privacy and security risks related to data sharing and hacking are also areas of concern [22]. Cost and accessibility may limit widespread adoption, as these devices can be expensive and may not be covered by insurance for all individuals. Lastly, the learning curve associated with using and interpreting data from these devices might pose challenges for some users, impacting their overall experience and effectiveness in diabetes management [23,24].

Understanding the influencing factors of CGM adoption holds paramount importance due to its potential impact on individual health outcomes and advancements in diabetes management technology. By identifying the drivers or barriers influencing the adoption of CGM systems, healthcare providers, policymakers, and technology developers gain critical insights. These insights aid in tailoring interventions, designing educational programs, and refining the technology itself to better meet the needs and preferences of individuals with diabetes. Improved adoption rates could lead to better glycemic control, reduced diabetes-related complications, and enhanced quality of life for those managing diabetes, underscoring the necessity of comprehensively studying these influencing factors. This cross-sectional study seeks to address this knowledge gap by examining the behavioral intentions (BIs) of wearable insulin biosensors among diabetes patients, the factors that drive or hinder their usage, and the implications for diabetes management and healthcare outcomes.

## Materials and methods

The present study used deductive quantitative cross-sectional approach to draw precise conclusions based on empirical evidence of various factors influencing the adoption of wearable inulin biosensors.

Recruitment and sampling

The participants in this study included diabetes patients recruited from public hospitals and social self-help communities. As participants are purposively recruited from the selected institutions, convenience and purposive sampling techniques were adopted [25]. The inclusion criteria included adult diabetes patients who have used or have been using wearable insulin biosensors for the management of their condition. Given the total number of diabetes patients in Saudi Arabia to be seven million in 2023 [26], the estimated sample size was calculated using Cochran's formula [27] (n=Z^2^ p(1-p)/e^2^, where “e” is error; “p” is population), which is identified to be 383, and the post-hoc power analysis resulted in 100% power.

Instruments

The survey questionnaire is partitioned into two distinct components. The initial phase of the study is dedicated to the acquisition of demographic data pertaining to age, gender, educational background, and prior experience with AI-enabled technologies. The subsequent phase of the study is dedicated to the acquisition of data pertaining to the many aspects that exert influence on AI technology. This study has incorporated four elements, namely performance expectancy (PE), effort expectancy (EE), social influence (SI), and facilitating conditions (FCs), as identified in previous studies [28,29]. Furthermore, the measurement of BI was derived from the work of [30]. Furthermore, the study incorporated three characteristics, namely perceived privacy risks (PPR), trust, and personal innovativeness (PI), as derived from the work of [31]. The questionnaire was developed on Google Forms, employing a hyperlink to facilitate access to the survey. A preliminary investigation was undertaken with a sample of 14 diabetic patients, and subsequent analysis was performed on the collected data. The Cronbach alpha coefficient was computed for all items and found to exceed 0.7 (Table 1), suggesting favorable internal consistency [32].

**Table 1:** Reliability of questionnaire items

	Definition	No. of items	Cronbach alpha
PE	Performance Expectancy refers to the user's belief concerning the extent to which using a particular technology will help them enhance their job performance or accomplish specific tasks more efficiently.	4	.826
EE	Effort expectancy refers to the user's perception of the ease of use and simplicity associated with utilizing a particular technology.	3	.912
SI	Social influence refers to the degree to which an individual perceives that others, such as friends, family, colleagues, or influential entities, influence their decision to adopt or use a particular technology.	3	.873
FCs	Facilitating conditions represent the degree to which individuals perceive the presence of technical support, resources, and infrastructure available to assist in the use and adoption of a specific technology.	4	.791
BI	Behavioral intentions refer to an individual's willingness or readiness to exert effort in adopting and using a specific technology.	3	.847
Trust	Trust refers to an individual's confidence, belief, or reliance on the credibility, integrity, and security aspects associated with a specific technology or system.	4	.811
PPR	Perceived Privacy Risks refer to an individual's subjective assessment or concerns regarding potential threats to their privacy associated with using a specific technology or system.	4	.739
PI	Personal innovativeness refers to an individual's inclination or predisposition toward adopting and using new technologies or innovative solutions.	4	.869

Ethical considerations

All the participants were fully informed about the study through an information sheet attached to the invitation email. An informed consent was taken from all the participants using a check button, before starting the survey. The participation was voluntary and the participants were assured of their anonymity and their rights with respect to the data. Ethical approval (IRB-2023-03-475) was received from the ethics committee at Imam Abdulrahman Bin Faisal University.

Data collection

A participant information sheet is attached along with the invitation email (containing a survey link), explaining the rights of the participants, and forwarded to all the patients who agreed to participate in the survey. A total of 274 patients participated in the survey. However, 28 responses were incomplete. After cleaning the data, a total of 248 patients' responses were considered for data analysis.

Data analysis

To attain the objectives of the research, the researcher utilized the statistical package for the Social Sciences (SPSS, Version 24, (IBM Corp., Armonk, NY)) to analyze the data. Descriptive statistics will be used to characterize the participants’ demographic data. In addition, a two-sample t-test with unequal variances for differences between gender groups, and single-factor Analysis of Variance (ANOVA) were used for analyzing the differences between age and education-based groups. Furthermore, Person correlation coefficients were used to compare the relationship between various factors.

## Results

The data from Table 2 reveal a nearly equal gender distribution, with 126 males (50.8%) and 122 females (49.2%) contributing to the study. Regarding age distribution, the majority fell within the age groups of 31-40 (38.7%) and 18-30 (28.6%), while smaller proportions were distributed among the 41-50 (22.6%) and >=51 (10.1%) categories. In terms of education, the largest group held a diploma (33.1%), followed by individuals with bachelor's degrees (31.5%) and master's degrees (21.0%). Notably, a smaller percentage of participants were either uneducated (4.8%) or held Ph.D. qualifications (1.2%). This diverse demographic representation within the study indicates a balanced gender ratio and a varied distribution across age and education levels, offering insights into potential factors influencing the adoption of wearable insulin biosensors among individuals with different backgrounds and characteristics.

**Table 2:** Participants' demographics

Variables		N	Relative frequency
Gender	Male	126	50.8%
	Female	122	49.2%
Age (in years)	18-30	71	28.6%
	31-40	96	38.7%
	41-50	56	22.6%
	>=51	25	10.1%
Education	Uneducated	12	4.8%
	Primary/secondary education	21	8.5%
	Diploma	82	33.1%
	Bachelor’s degree	78	31.5%
	Master’s degree	52	21.0%
	Ph.D.	3	1.2%

**Figure 1:**
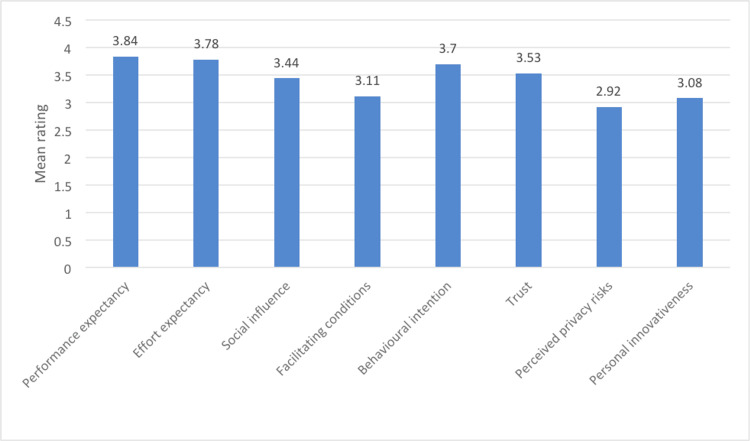
Mean ratings of various factors influencing adoption of wearable insulin biosensors

Figure 1 presents mean ratings on a scale of 1 to 5 for various influencing factors affecting the adoption of wearable insulin biosensors. PE was rated the highest at 3.84, indicating that participants generally strongly agreed with the idea that using these biosensors would enhance their performance in managing diabetes. EE followed closely at 3.78, suggesting a relatively high level of agreement that using the biosensors would not demand excessive effort. SI, with a mean rating of 3.44, indicated moderate agreement regarding the impact of social factors on adoption. FCs received a lower mean rating of 3.11, possibly suggesting that participants were somewhat less confident about the presence of adequate resources and support for adoption. BI scored 3.7, indicating a reasonably positive attitude towards adopting the biosensors. Trust in the technology received a mean rating of 3.53, suggesting a moderate level of trust. On the other hand, PPRs scored relatively lower at 2.92, indicating some concern among participants regarding privacy issues associated with these biosensors. Finally, PI scored 3.08, suggesting a moderate level of agreement regarding the participants' personal inclination towards adopting innovative technologies. Overall, these ratings depict generally positive attitudes towards performance and ease of use but also indicate some reservations concerning privacy and the availability of FCs for adoption.

Table 3 illustrates differences among participant groups concerning various factors related to the adoption of wearable insulin biosensors, including PE, EE, SI, and FCs. Significant variations were observed across genders, age groups, and education levels. Regarding gender, males scored notably higher than females across all factors, with statistically significant differences (p < 0.05). In terms of age, participants aged 18-30 exhibited higher mean scores for PE compared to other age groups, with a statistically significant difference (p < 0.05) observed. However, no significant differences were found for EE, SI, and FCs across age groups. Concerning education, individuals with uneducated or primary/secondary education levels showcased lower mean scores across all factors compared to those with higher education levels, but these differences were not statistically significant (p > 0.05), except for the “Others” category, which displayed mixed educational backgrounds. These findings suggest that gender and age could significantly influence the perception of various factors affecting the adoption of wearable insulin biosensors, while the impact of education level appears less pronounced, except for certain distinct educational backgrounds represented within the “Ph.D.” category.

**Table 3:** Differences among the participants groups with respect to performance expectancy, effort expectancy, social influence, and facilitating conditions *: Statistically significant difference at 0.05 CI

Variables		N	Performance expectancy		Effort expectancy		Social influence		Facilitating conditions	
			Mean	P-value	Mean	P-value	Mean	P-value	Mean	P-value
Gender	Male	126	4.05	.0032*	4.00	.0041*	3.63	.0046*	3.32	.0026*
	Female	122	3.62		3.55		3.25		2.89	
Age (in years)	18-30	71	4.05	.0024*	3.89	.1292	3.52	.1691	3.15	.4317
	31-40	96	4.02		3.89		3.54		3.22	
	41-50	56	3.47		3.66		3.34		2.93	
	>=51	25	3.39		3.29		3.05		2.97	
Education	Uneducated	12	4.21	.442	4.17	.2135	3.53	.2553	3.21	.0821
	Primary/secondary education	21	3.67		3.41		3.25		2.89	
	Diploma	82	3.87		3.82		3.52		3.20	
	Bachelor’s degree	78	3.96		3.94		3.59		3.32	
	Master’s degree	52	3.60		3.51		3.15		2.75	
	Ph.D	3	3.92		4.11		3.44		2.67	

Table 4 presents variations among participant groups concerning BI, Trust, PPR, and PI in adopting wearable insulin biosensors. The data highlights several significant differences based on gender, age, and education levels. In terms of gender, males generally exhibited higher mean scores compared to females for Trust and PI, with statistically significant differences (p < 0.05). However, no significant differences were observed for BIs and PPRs across genders. Regarding age groups, participants aged 18-30 demonstrated higher mean scores for PI compared to older age brackets, with a statistically significant difference (p < 0.05). However, no significant differences were found for BI, Trust, and PPR across age groups. Concerning education, participants with higher educational levels, such as bachelor's and Ph.D., displayed higher mean scores for Trust and PI, with statistically significant differences (p < 0.05) observed for several categories. Additionally, those with uneducated or primary/secondary education exhibited higher mean scores for PPR compared to individuals with higher educational qualifications, with significant differences noted (p < 0.05). These findings suggest that gender, age, and education levels play distinct roles in influencing trust, PI, and PPRs associated with the adoption of wearable insulin biosensors, highlighting specific demographic differences in attitudes and perceptions towards this technology.

**Table 4:** Differences among the participants groups with respect to behavioral intention, trust, perceived privacy risk, and personal innovativeness *: Statistically significant difference at 0.05 CI

Variables		N	Behavioral intentions		Trust		Perceived privacy risks		Personal innovativeness	
			Mean	P-value	Mean	P-value	Mean	P-value	Mean	P-value
Gender	Male	126	3.81	.1522	3.72	.0037*	3.03	.0726	3.28	.0003*
	Female	122	3.59		3.33		2.80		2.88	
Age (in years)	18-30	71	3.79	.41	3.64	.2691	3.00	.4465	3.11	.0386*
	31-40	96	3.78		3.60		2.90		3.24	
	41-50	56	3.57		3.39		2.76		2.87	
	>=51	25	3.43		3.24		3.11		2.84	
Education	Uneducated	12	3.94	.0934	3.98	.0183*	3.44	.0002*	3.35	.0047*
	Primary/secondary education	21	3.25		3.07		2.23		2.89	
	Diploma	82	3.80		3.59		2.91		3.13	
	Bachelor’s degree	78	3.89		3.72		3.21		3.30	
	Master’s degree	52	3.40		3.23		2.69		2.70	
	Ph.D.	3	3.67		3.25		2.25		2.92	

Table 5 displays Pearson's correlations between various factors related to the adoption of wearable insulin biosensors. BI exhibits strong positive correlations with several influencing factors. Notably, BI demonstrates robust positive correlations with PE, EE, SI, and Trust, all ranging from 0.808 to 0.877. This suggests that as individuals perceive higher performance expectations, ease of use, SI, and trust in the technology, their BIs to adopt these biosensors also tend to increase significantly. Additionally, BI shows a moderate positive correlation with FCs at 0.763, indicating that when participants perceive adequate resources and support for adoption, their intentions to adopt the technology also tend to strengthen.

**Table 5:** Correlations between various factors

	PE	EE	SI	FCs	BI	Trust	PPR	PI
PE	1							
EE	0.909	1						
SI	0.830	0.908	1					
FCs	0.681	0.751	0.885	1				
BI	0.808	0.872	0.877	0.763	1			
Trust	0.800	0.863	0.833	0.828	0.866	1		
PPR	0.335	0.337	0.332	0.374	0.419	0.431	1	
PI	0.542	0.581	0.676	0.798	0.594	0.674	0.617	1

However, PPRs exhibit relatively weaker correlations with BI and other factors, signifying that concerns about privacy risks might not strongly influence participants' intentions to adopt these biosensors compared to other influencing factors. Overall, these correlations underscore the pivotal role of PE, EE, SI, Trust, and to a slightly lesser extent, FCs, in shaping individuals' BIs toward adopting wearable insulin biosensors, emphasizing the importance of these factors in influencing adoption decisions.

## Discussion

The purpose of this study is to analyze the BIs of wearable insulin biosensors among diabetes patients, and the factors that drive or hinder their adoption. Accordingly, the factors including PE and EE were identified to be high among the participants reflecting the improved performance and reduced efforts in managing diabetes, which may lead to improved BIs in the adoption of insulin biosensors. However, PPRs, FCs, and PI reflect issues with security and privacy and lack of adequate resources to support the adoption that might have led to low inclination and trust towards the insulin biosensors. Factors, such as PE, EE, and FCs, were found to be positively influencing the adoption of similar mHealth technologies for diabetes management in previous research [33,34]. However, recent studies focusing on the influencing factors of wearable devices for diabetes and other conditions management have observed that although PE, EE, and BI were high; FCs, PPR, SI, and PI were found to be of low or negative impact on the intentions to adopt wearable devices [35-38]. Issues, such as privacy and security concerns, have significantly influenced the user's trust in wearable devices, which had an indirect impact on the adoption of wearable devices.

Furthermore, the influence of demographic variables is evident from the results. It has been observed that male participants rated all the factors high compared to the female participants except BI, where both genders exhibited similar intentions. This difference is more evident in relation to PE, EE, and PI, indicating that male participants perceived wearable devices to be more effective in improving performance, and easy to use leading to greater inclination compared to female participants. Statistically significant differences were observed in relation to younger participants who found PE and PI to be more compared to younger participants, indicating the differences in the inclinations towards innovative technologies like wearable devices. These findings are similar to a recent study [39], which has identified PE and PI to be low among the elderly population in relation to wearable health devices. Furthermore, it is interesting to observe that less educated participants' PPRs were high compared to highly educated; but they also had high levels of trust compared to highly educated participants, indicating the complex nature of perceptions among the education-based groups in relation to trust, PPR, and PI. The results from previous research reflected varying findings in relation to the impact of socio-demographic characteristics on the influencing factors. For instance, a recent study in China [40] has found that Socio-demographic characteristics including gender, age, and education did not exert a significant direct influence on adoption intention; whereas another similar study in the USA [41] has identified a significant impact of Socio-demographic characteristics and cultures on adoption intentions. Accordingly, the findings in this study have identified that PE, EE, and trust are positively related to adoption intentions, while PPR and PI exhibited weak relations with adoption intentions. Therefore, it can be assumed that the perceptions of diabetes patients with respect to wearable devices may be influenced by socio-demographic, economic, cultural, and regional factors; highlighting the need for extensive research to better understand the influencing factors of wearable devices such as insulin biosensors and their impact on adoption intentions.

Implications

The findings of this study on the adoption of wearable insulin biosensors among diabetes patients carry both practical and theoretical implications. From a practical standpoint, understanding the factors influencing adoption intentions, such as PE, EE, trust, and FCs, offers crucial insights for healthcare providers, policymakers, and technology developers. These insights can aid in tailoring interventions, designing educational programs, and refining the technology itself to better meet the needs and preferences of individuals with diabetes. For instance, focusing on enhancing user trust through transparent and secure design features, ensuring ease of use, and improving access to necessary resources could potentially increase the adoption rates of wearable insulin biosensors. The study underscores the importance of addressing concerns related to privacy risks and the need for adequate supporting infrastructure to facilitate the uptake of such innovative technologies in diabetes management. Theoretical implications lie in enriching our understanding of how socio-demographic factors such as gender, age, and education influence perceptions of technology adoption. These insights contribute to the broader theoretical framework concerning technology acceptance models by highlighting the nuanced roles of various influencing factors and demographic characteristics in shaping individuals' BIs toward adopting innovative healthcare technologies. Ultimately, bridging the gap between theoretical insights and practical applications could significantly impact the uptake of wearable insulin biosensors, potentially improving diabetes management and overall healthcare outcomes for affected individuals.

Limitations

There are a few limitations that can be observed in this study. Firstly, the study's reliance on a cross-sectional design limits its ability to establish causal relationships between variables, offering snapshots of associations at a single point in time. Longitudinal studies could provide more robust insights into how factors influencing adoption intentions might evolve over time. Additionally, the study's sample primarily comprised participants from public hospitals and self-help communities, potentially introducing selection bias and limiting the generalizability of findings to broader populations with diverse healthcare access or socioeconomic backgrounds. The self-reported nature of data collection via survey questionnaires might also introduce response bias or socially desirable responses, affecting the accuracy and reliability of the gathered information. Furthermore, while the study explores various influencing factors, it might have overlooked additional variables that could impact adoption intentions, such as cultural influences or prior experiences with similar technologies. Addressing these limitations could strengthen the study's validity and provide a more comprehensive understanding of the complexities surrounding the adoption of wearable insulin biosensors in diabetes management.

## Conclusions

In conclusion, this study sheds light on the multifaceted landscape surrounding the adoption of wearable insulin biosensors among individuals managing diabetes. The findings underscore the significance of factors like PE, EE, trust, and FCs in shaping BIs toward embracing this innovative technology. Practical implications emphasize the need for tailored interventions focusing on enhancing user trust, ensuring ease of use, and addressing concerns related to privacy risks to facilitate wider acceptance and uptake of wearable insulin biosensors in diabetes management. Additionally, the study's theoretical implications emphasize the intricate interplay between demographic factors and technology acceptance, contributing to the broader understanding of adoption behavior within the realm of healthcare technologies. Despite certain limitations inherent in the study design and sampling, the insights gleaned serve as a foundation for further exploration, highlighting the necessity for ongoing research to better comprehend the evolving dynamics influencing the integration of novel healthcare technologies into the lives of individuals managing chronic conditions like diabetes. Ultimately, addressing these insights could pave the way for more effective, personalized, and accessible solutions, thereby potentially improving the quality of care and outcomes for those living with diabetes.
